# Standardized flattening filter free volumetric modulated arc therapy plans based on anteroposterior width for total body irradiation

**DOI:** 10.1002/acm2.12827

**Published:** 2020-02-11

**Authors:** Rebecca Frederick, Alana Hudson, Alex Balogh, Jeffrey Q. Cao, Greg Pierce

**Affiliations:** ^1^ Department of Medical Physics Tom Baker Cancer Centre Calgary AB Canada; ^2^ Department of Physics and Astronomy University of Calgary Calgary AB Canada; ^3^ Department of Oncology University of Calgary Calgary AB Canada; ^4^ Division of Radiation Oncology Tom Baker Cancer Centre Calgary AB Canada

**Keywords:** flattening filter free, total body irradiation, treatment planning, treatment technique comparison, volumetric modulated arc therapy

## Abstract

In this work, the feasibility of using flattening filter free (FFF) beams in volumetric modulated arc therapy (VMAT) total body irradiation (TBI) treatment planning to decrease protracted beam‐on times for these treatments was investigated. In addition, a methodology was developed to generate standardized VMAT TBI treatment plans based on patient physical dimensions to eliminate plan optimization time. A planning study cohort of 47 TBI patients previously treated with optimized VMAT ARC 6 MV beams was retrospectively examined. These patients were sorted into six categories depending on height and anteroposterior (AP) width at the umbilicus. Using Varian Eclipse, clinical 40 cm × 10 cm open field arcs were substituted with 6 MV FFF. Mid‐plane lateral dose profiles in conjunction with relative arc output factors (RAOF) yielded how far a given multileaf collimator (MLC) leaf must move in order to achieve a mid‐plane 100% isodose for a specific control point. Linear interpolation gave the dynamic MLC aperture for the entire arc for each patient AP width category, which was subsequently applied through Python scripting. All FFF VMAT TBI plans were then evaluated by two radiation oncologists and deemed clinically acceptable. The FFF and clinical VMAT TBI plans had similar Body–5 mm D98% distributions, but overall the FFF plans had statistically significantly increased or broader Body–5 mm D2% and mean lung dose distributions. These differences are not considered clinically significant. Median beam‐on times for the FFF and clinical VMAT TBI plans were 11.07 and 18.06 min, respectively, and planning time for the FFF VMAT TBI plans was reduced by 34.1 min. In conclusion, use of FFF beams in VMAT TBI treatment planning resulted in dose homogeneity similar to our current VMAT TBI technique. Clinical dosimetric criteria were achieved for a majority of patients while planning and calculated beam‐on times were reduced, offering the possibility of improved patient experience.

## 1 INTRODUCTION

Total body irradiation (TBI) has a prominent role in the treatment of a variety of blood disorders requiring hematopoietic stem cell transplantation (HSCT), including cancers such as leukemia and lymphoma.^1^, ^2^, ^3^, ^4^, ^5^ The use of TBI in conjunction with chemotherapy for conditioning prior to HSCT has been shown to be clinically beneficial as TBI is able to target sanctuary sites such as the brain and testes, and aids in suppressing the immune system to improve the chances of successful stem cell engraftment.^1^, ^4^, ^5^ TBI requires the delivery of a homogeneous dose of radiation to the entire body to target all malignant blood cells as well as the bone marrow where most blood cells are formed.

Recently, one focus of TBI technique development has been improving planning accuracy and decreasing toxicities associated with myeloablative therapy. This includes the use of volumetric modulated arc therapy (VMAT), which has shown promise in improving dose homogeneity and reducing the dose to organs at risk (OAR). Springer et al developed a 9‐15 isocenter VMAT technique where the patient is longitudinally translated to capture the entire planning target volume (PTV) in eight segments.^6^ Smooth junctions between segments were achieved using inverse planning, resulting in a homogeneous dose delivery. A similar technique was described by Tas et al, but only 3‐5 isocenters were required.^7^ They additionally completed three‐dimensional dose‐volume histogram (DVH) based quality assurance of their plans including their junction regions, thus presenting a robust and accurate method of TBI delivery. While VMAT TBI is resulting in higher quality plans, some problems persist. Moving to optimized plans requires an increase in treatment planning resources. For the higher complexity VMAT TBI techniques, the contouring time can be up to six hours, and treatment planning time over a day.^6^ In addition, highly modulated plans increase the already prolonged beam‐on times for TBI treatments. Reported times including patient set‐up for one fraction VMAT TBI are between 55 and 120 min for adult patients.^6^, ^7^ Multi‐fraction treatments lead to considerable clinic resource utilization as well as the patient spending long periods on the treatment couch resulting in heightened discomfort.

In an effort to decrease the amount of time required for VMAT TBI while attaining similar dose homogeneity, we present a treatment planning study investigating the feasibility of a VMAT TBI technique utilizing flattening filter free (FFF) beams. FFF beams permit a higher dose rate delivery, so this modality substitution should result in decreased beam‐on times and overall shorter treatments. In addition, the peaked dose profile for FFF beams provides the possibility of a body contour match at the umbilicus level in comparison to the standard flattened dose profile, with subsequent isodose lines that are flatter prior to multileaf collimator (MLC) modulation. To further improve time efficiency, a set of standardized plans with forward‐planned MLC leaf positions are presented that are suitable for patients with certain physical dimensions, lessening the strain on planning resources. A discussion of potential lung toxicities with an increased dose rate then follows.

## 2 MATERIALS AND METHODS

### 2.1 Patient stratification

Seventy‐two adult patients who underwent HSCT at the Tom Baker Cancer Centre (TBCC) in Calgary, Canada between January 2016 and April 2017 were prescribed TBI with a total dose of 4 Gy delivered in 2 Gy per fraction b.i.d. with at least six hours between fractions. Various diseases were treated, including but not limited to acute myeloid leukemia (AML), acute lymphoblastic leukemia (ALL), and myelodysplastic syndrome (MDS). CT data were retrospectively examined to determine height and anteroposterior (AP) width at the level of the umbilicus, with the median (range) being 181 cm (162‐210) and 22.4 cm (16.0‐37.4), respectively. Patients were categorized by AP width, with each category covering a 2 cm range. Four categories with a total of 47 patients were selected to be part of the study, covering the range of widths most frequently seen at the TBCC and the suggested range for adult patients in the American Association of Physicists in Medicine (AAPM) Report 17^4^: 17‐19, 19‐21, 21‐23, and 23‐25 cm. This AP width range was additionally chosen as there were at least five patients in each category, which was sufficient to develop and test the standardized TBI plans. The last two categories were further divided into short and tall subsets, each approximately covering a 20 cm range. Height and width distribution across the categories are described in Table 1.

**Table 1:** Summary of patient categorizations and the corresponding distribution of heights and AP widths. Values are reported as median (range) where applicable.

Category	Number of patients	Anteroposterior width at umbilicus (cm)	Height (cm)
17‐19 cm	8	18.4 (16.8‐19.0)	174 (163‐182)
19‐21 cm	10	20.2 (19.3‐21.0)	179 (173‐204)
21‐23 cm Short	8	21.8 (21.1‐22.4)	175 (162‐181)
21‐23 cm Tall	10	21.4 (21.2‐22.8)	190 (185‐202)
23‐25 cm Short	6	24.1 (23.4‐24.5)	177 (162‐180)
23‐25 cm Tall	5	24.0 (23.2‐24.5)	199 (186‐210)

### 2.2 Standard FFF VMAT plan development

#### 2.2.1 Current clinical treatment technique

**Figure 1:**
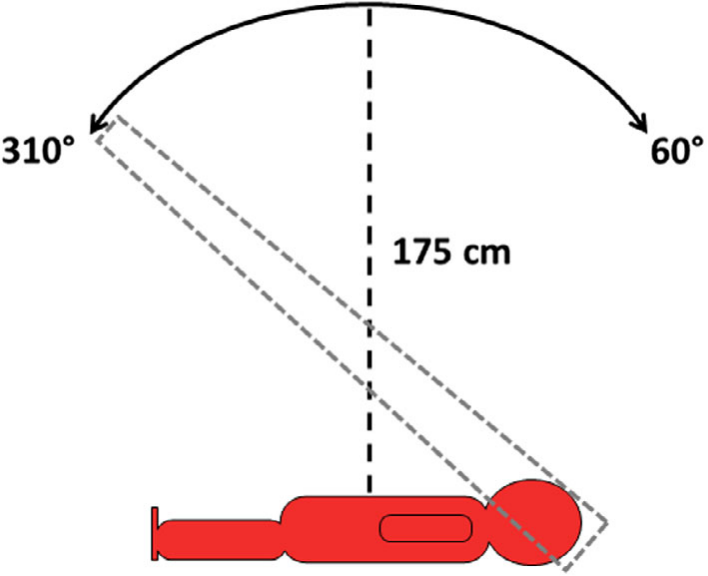
A schematic of the clinical VMAT TBI technique used as the basis of the FFF VMAT TBI technique. The gantry arcs over the patient 7‐8 times between gantry angles 310° and 60° (IEC 601) with the patient at an extended SSD of approximately 175 cm. The patient is treated in both the supine (shown) and prone orientations. FFF, flattening filter free; TBI, total body irradiation; VMAT, volumetric modulated arc therapy.

All 47 patients were previously treated using the TBCC's clinical VMAT TBI technique described in detail elsewhere and summarized here.^8^ This anterior‐posterior (AP/PA) technique utilizes both supine and prone patient orientations at an extended source‐to‐surface distance (SSD) of approximately 175 cm, a custom bed oriented at 90° (IEC 601), and a beam spoiler. A 6 MV single‐isocenter sweeping arc between gantry angles 310° and 60° (IEC 601) was developed with control point weighting to account for inverse square law (ISL) effects and spoiler attenuation, similar to work done by Jahnke et al.^9^ The linear accelerator (linac) gantry arcs over the patient 7‐8 times per setup orientation in order to deliver the required monitor units (MU) with a nominal dose rate of 600 MU/min. A schematic of the technique is illustrated in Fig. 1. Patient CT data are used for treatment planning in Eclipse (Varian Medical Systems, Palo Alto, CA). Patient planning CTs are taken head‐first supine with a slice thickness of 0.5 cm and are only maximum ~135 cm in length; the most inferior slice around mid‐thigh is replicated to simulate the patient's legs and reproduce the entire patient length. Contoured structures include the lungs, and the Body contour retracted 5 mm from the skin (Body–5 mm). The clinical technique also makes use of minor MLC modulation to shield the lungs and improve dose homogeneity, provided by the progressive resolution optimizer (PRO) for VMAT planning in Eclipse.

#### 2.2.2 FFF delivery technique

The clinical VMAT TBI technique was used as a template for the development of the FFF delivery technique. Patient setup, orientation, and immobilization were kept the same for the FFF VMAT TBI technique. Two major changes to the clinical technique were made: the beam energy was changed from 6 MV to 6 MV FFF, and the MLC shapes were pregenerated using custom code to achieve the desired dose distribution within the body, resulting in a set of standard treatment plans. The process of standard plan generation follows.

##### Relative arc output factors (RAOF)

FFF plan construction began with a 310°‐60° 40 cm × 10 cm open field arc with meterset weights based off of the clinical VMAT TBI technique, summarized in Table 2. The beam energy was 6 MV FFF, the nominal dose rate 1400 MU/min, and dose was calculated in Eclipse using the analytical anisotropic algorithm (AAA) version 11.0.31 with grid size 0.5 cm.

**Table 2:** Meterset weights used for all FFF VMAT TBI plans, and the resulting gantry speeds for a delivery of 700 monitors units per arc.

Angle range (°)	Meterset weight	Gantry speed (°/s)
310‐320	2.250	2.583
320‐330	1.750	3.321
330‐340	1.229	4.729
340‐350	1.074	5.412
350‐10	1.000	5.813
10‐20	1.200	4.844
20‐30	1.300	4.471
30‐40	1.500	3.875
40‐50	2.000	2.906
50‐55	2.667	2.179
55‐60	3.780	1.538

Abbreviations: FFF, flattening filter free; VMAT, volumetric modulated arc therapy; TBI, total body irradiation.

**Figure 2:**
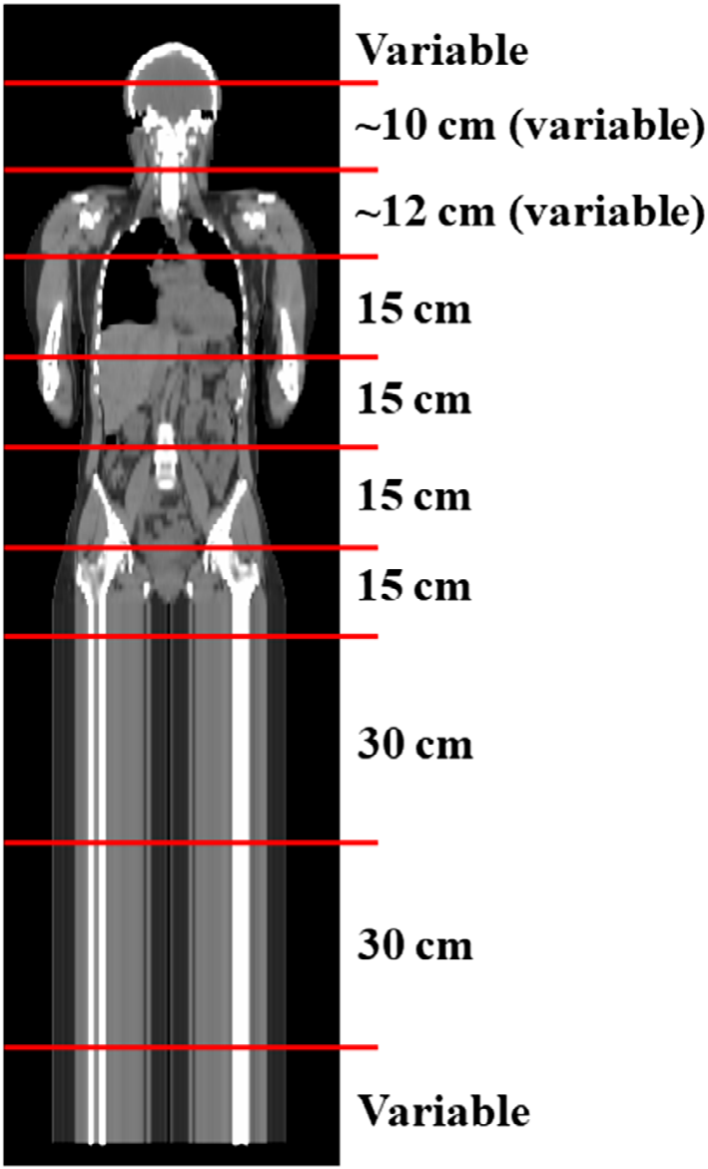
A coronal patient CT image with the nine cranial‐caudal locations where lateral dose profiles were collected to compute MLC modulation indicated by horizontal lines. Spacing between locations is indicated to the right of the image. MLC, multileaf collimator.

**Figure 3:**
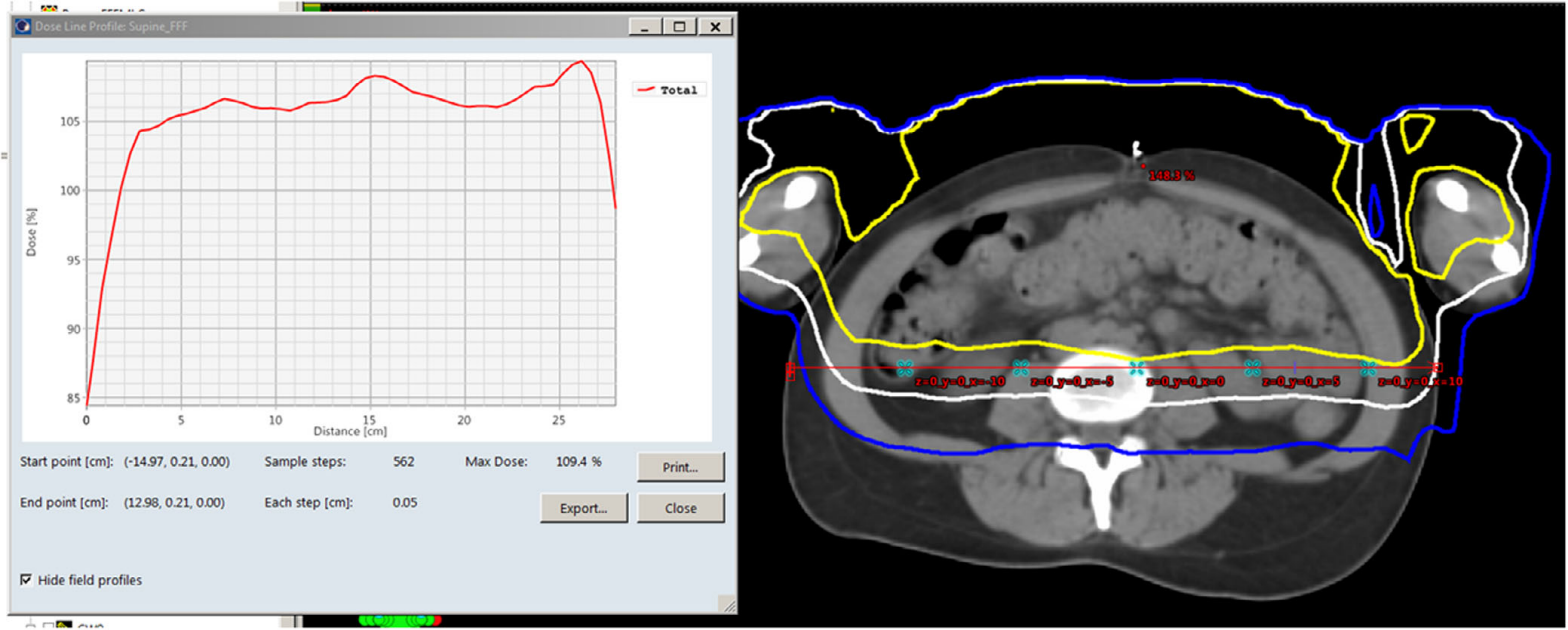
A screen capture from Eclipse of an axial CT slice of a patient at the level of the umbilicus with a supine open field 6 MV FFF VMAT TBI plan applied. On the right is the CT image with the 110% isodose level in yellow, the 100% isodose level in white, and the 90% isodose level in blue. The mid‐plane red line on the CT image indicates the location of the dose profile, with the profile itself presented on the left. The crosses along the mid‐plane red line on the CT image are reference points. FFF, flattening filter free; TBI, total body irradiation; VMAT, volumetric modulated arc therapy.

The goal is to produce a dose distribution that is flat in both the lateral and cranial‐caudal directions at a mid‐plane depth, with 100% of the dose for each patient orientation at mid‐plane, allowing for full even coverage when combined with both orientations. To assess the homogeneity of the dose distribution, discrete lateral dose profiles in the patient's AP mid‐plane were collected for both the supine and prone orientations at 9‐10 cranial‐caudal locations for three patients from each category, and then averaged patient‐wise. Profiles were discretized using reference points laterally spaced by 5 cm. Lateral dose profiles were longitudinally spaced by approximately 15 cm with the exception of the cranial region where profiles were typically closer spaced due to large variation in body contour (ranging between 10 and 16 cm) and the simulated legs where less profiles were required. Spacing was kept as consistent as possible between patients. One of the cranial‐caudal locations was chosen to be the lungs to provide shielding. An example of the cranial‐caudal spacing is shown in Fig. 2, and a lateral dose profile in Fig. 3.

After collecting the open field dose values, a method was developed to change the field size to achieve a flat 100% isodose line at mid‐plane. The factor required to change the mid‐plane profile isodose values to 100% was calculated for each point as follows:

(1) $$\text{RAOF}\;=\;\frac{100\%}{\text{Open Field Isodose \textbackslash{}\%}\;}$$

**Figure 4:**
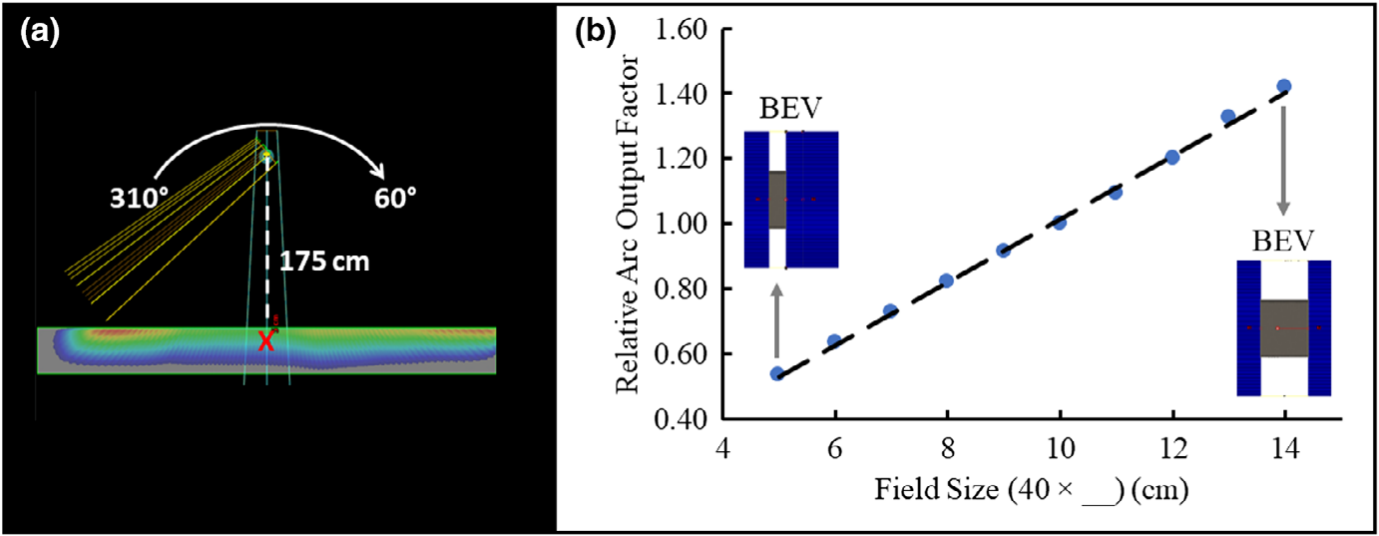
An illustration of the Eclipse setup and the RAOF curve. (a) RAOFs were modeled in a simulated 20 cm × 30 cm × 200 cm water phantom at an extended SSD of 175 cm in Eclipse. Arc energy, angles, and meterset weights matched those used for the FFF VMAT TBI plans. RAOFs for MLC‐defined fields between 40 cm × 5 cm and 40 cm × 14 cm were determined at 5 cm depth directly below isocenter (red cross). (b) The RAOF curve is normalized to a field size of 10 cm. The x‐axis describes the change in field size associated with the MLC. The Y‐jaw was static at 40 cm. The dashed line illustrates the linear fit used for MLC modulation calculation. Two beam's eye view (BEV) images of the MLC‐defined aperture are shown for the 40 cm × 5 cm and 40 cm × 14 cm measurements. FFF, flattening filter free; MLC, multileaf collimator; RAOF, relative arc output factor; TBI, total body irradiation; VMAT, volumetric modulated arc therapy.

**Figure 5:**
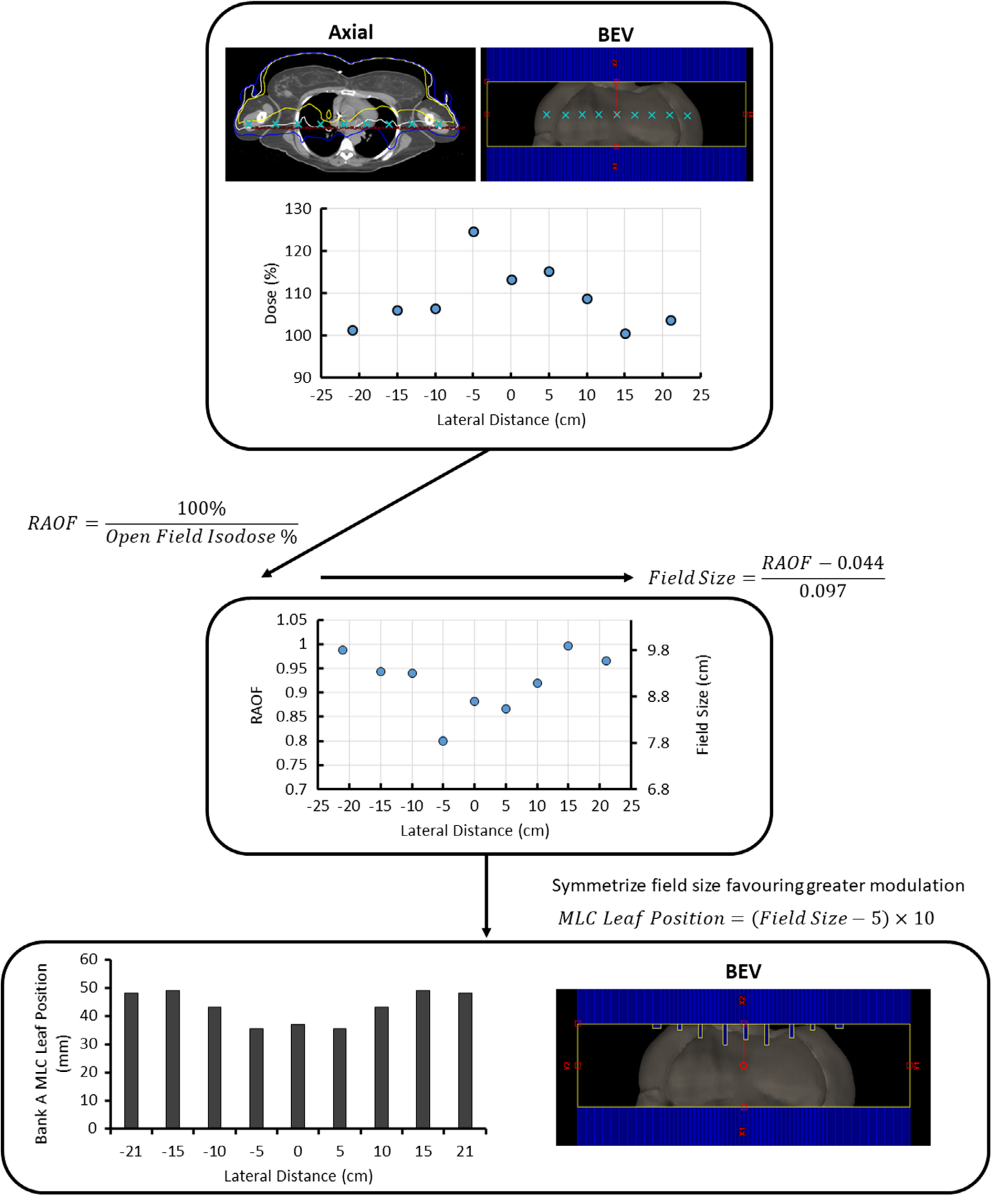
An example of MLC leaf position calculation at a single cranial‐caudal location. A discrete lateral dose profile as shown in the axial and beam's eye view (BEV) images is averaged from the dose data of three patients from each category. The RAOF equation (Equation 1) is then applied to each point. The required field size is then calculated at each point using the linear fit from Fig. 4 (Equation 2). In order to keep the standard plans more generalized, the field size is symmetrized over the patient's lateral mid‐line (x = 0 cm), favoring the larger field size changes. Finally, the MLC leaf position coded into the plan DICOM file is calculated. These positions correspond to those in the BEV shown next to the bar graph. MLC, multileaf collimator; RAOF, relative arc output factor.

Open Field Isodose % is the isodose value at a particular point on the profile described as a percentage of the prescription dose, and RAOF is the relative arc output factor. To deduce the necessary change in field size to adjust the isodose value for each calculated RAOF from the patient data, an RAOF function was generated from simulated data in Eclipse. A 20 cm × 30 cm × 200 cm water phantom was placed at an extended SSD of 175 cm. Dose was calculated using the open field arc and various field sizes defined by the MLCs to find output factors at 5 cm depth directly below isocenter. The Eclipse setup and the dose data normalized to a field size of 40 cm × 10 cm are illustrated in Fig. 4. Linear regression of this data allows mapping of RAOF values from patient data to corresponding field size changes. Thus, how far a given MLC leaf should move was approximated by substituting every RAOF value into the linear fit (Equation 2), yielding the corresponding field size change at that particular point. This process is outlined in Fig. 5, and is repeated for both the supine and prone orientations.

(2) $$\text{MLC Leaf Position}=\frac{\text{RAOF}-0.044}{0.097}$$

##### Standard FFF plan creation

**Figure 6:**
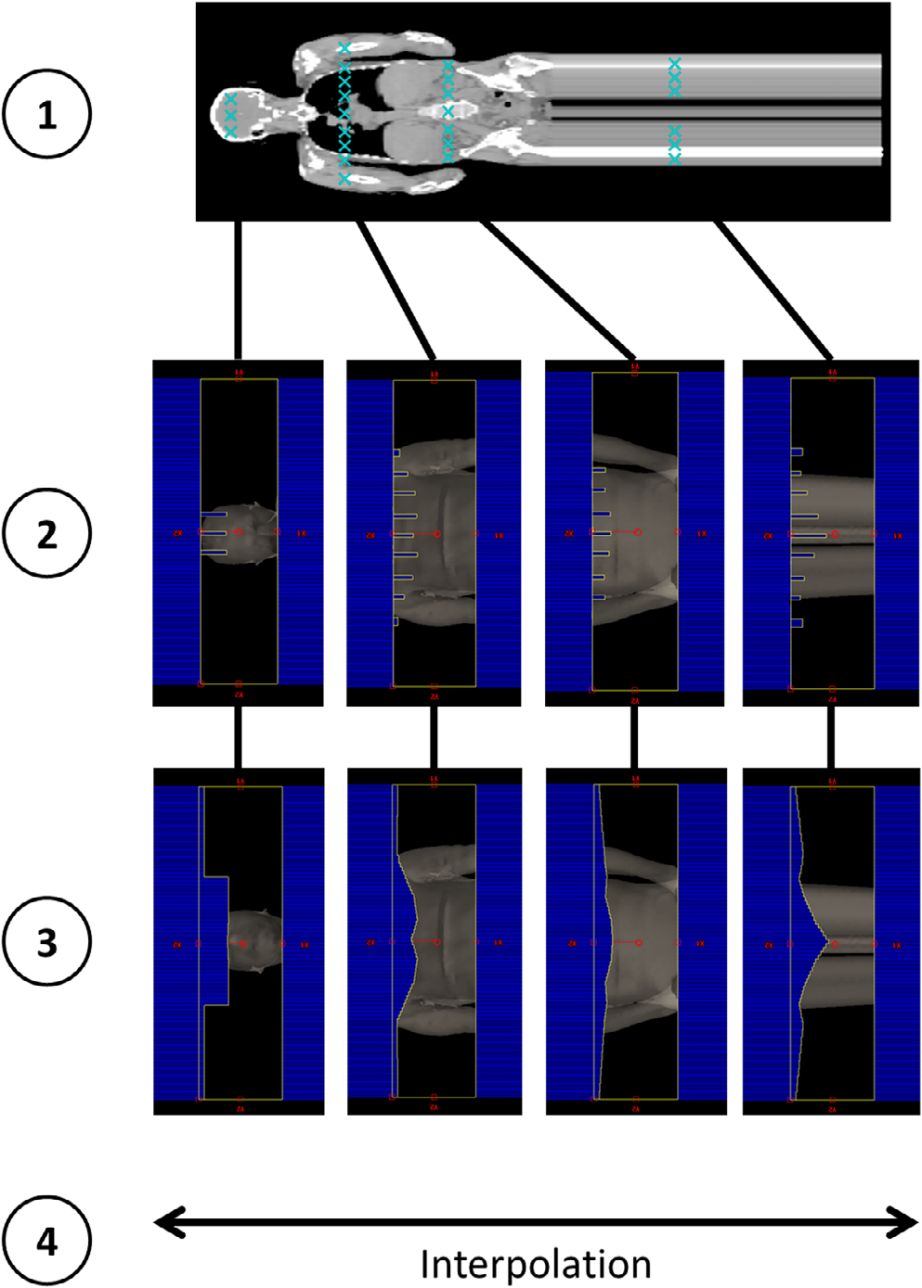
An illustration of the process of MLC aperture generation at four example cranial‐caudal locations. Step 1 is the collection of discrete AP mid‐plane dose profiles from the four locations. Application of the RAOF curve and trigonometric translation for the discrete profiles results in the beam's eye view MLC apertures shown in Step 2. The final MLC positions in Step 3 are produced by laterally interpolating the shapes from Step 2. A full VMAT arc is achieved by interpolating between the apertures shown in Step 3. MLC, multileaf collimator; RAOF, relative arc output factor; VMAT, volumetric modulated arc therapy.

The MLC leaf position for a given cranial‐caudal and lateral location at extended SSD was translated into a gantry angle (control point) and MLC leaf number. The RAOF‐calculated MLC leaf positions were linearly interpolated laterally to give the entire MLC shape for each of these control points, as shown in Fig. 6. The MLC leaf positions in between these discrete control points were planned through linear interpolation between the 9‐10 cranial‐caudal locations. In all cases, only one bank of MLCs was modulated, with the other remaining static at the largest required X2 jaw setting (5.0‐5.2 cm). Python (Python Software Foundation) scripting was used to write MLC leaf positions to a DICOM file. Examples of MLC positions used are shown in Fig. 6. For each AP width category, two DICOM files were created, corresponding to the supine and prone orientations. This resulted in a total of 12 standard plans, and all 47 patients had a supine and prone plan applied from this set depending on their categorization.

The DICOM files corresponding to the patient's AP width category were imported into Eclipse to yield a single VMAT arc for each orientation. The single VMAT arc was copied eight times for each patient orientation to make the final 16‐arc generated FFF TBI plan (fffTBI). Dose was normalized for each patient such that the Body–5 mm D98% of the FFF VMAT TBI plan was close to that of the existing clinical plan. DVH parameters corresponding to dose homogeneity, D98% and D2% of the Body–5 mm, as well as the mean lung dose (MLD) for the supine and prone plan sum were compared between the fffTBI and the existing clinical VMAT TBI plans (clinicalTBI) for each patient. The homogeneity index (HI), defined as the ratio of the D2% and D98%, is also compared for both sets of plans. This analysis was performed using the Wilcoxon Rank Sum test with a two‐tailed significance level of α = 0.05.

##### Plan evaluation

All fffTBI were reviewed by two radiation oncologists. Conservative dosimetric criteria (as a percentage of the prescription dose) that were used for plan evaluation include: Body–5 mm D98% ≥95%, Body–5 mm D2% ≤115%, and MLD ≤105%. The criterion that takes precedence is target coverage (D98%). An ALARA perspective was considered for the hotspot (D2%) and MLD objectives as they were more difficult to meet in most cases for both the fffTBI and clinicalTBI.

### 2.3 FFF plan robustness to set‐up errors

Testing the performance of the fffTBI with systematic set‐up errors was completed using Eclipse. Three patients with variable heights and widths from each category were selected for examination, for a total of 18 patients. Each arc isocenter was manually shifted 2 cm in the lateral, cranial‐caudal, and anteroposterior directions, and in both the positive and negative directions for these axes. The shifts were completed for both the supine and prone plans. Dose was recalculated for each separate shift using the same monitor units (MU) as the original fffTBI, and then a plan sum of the supine and prone plans corresponding to the same shift were completed to assess maximum error. The DVH parameters of the D98% and D2% of the Body–5 mm structure and MLD were collected, and the difference between the shifted FFF plan parameter and the original FFF plan parameter was taken.

## 3 RESULTS

### 3.1 Dosimetry

**Figure 7:**
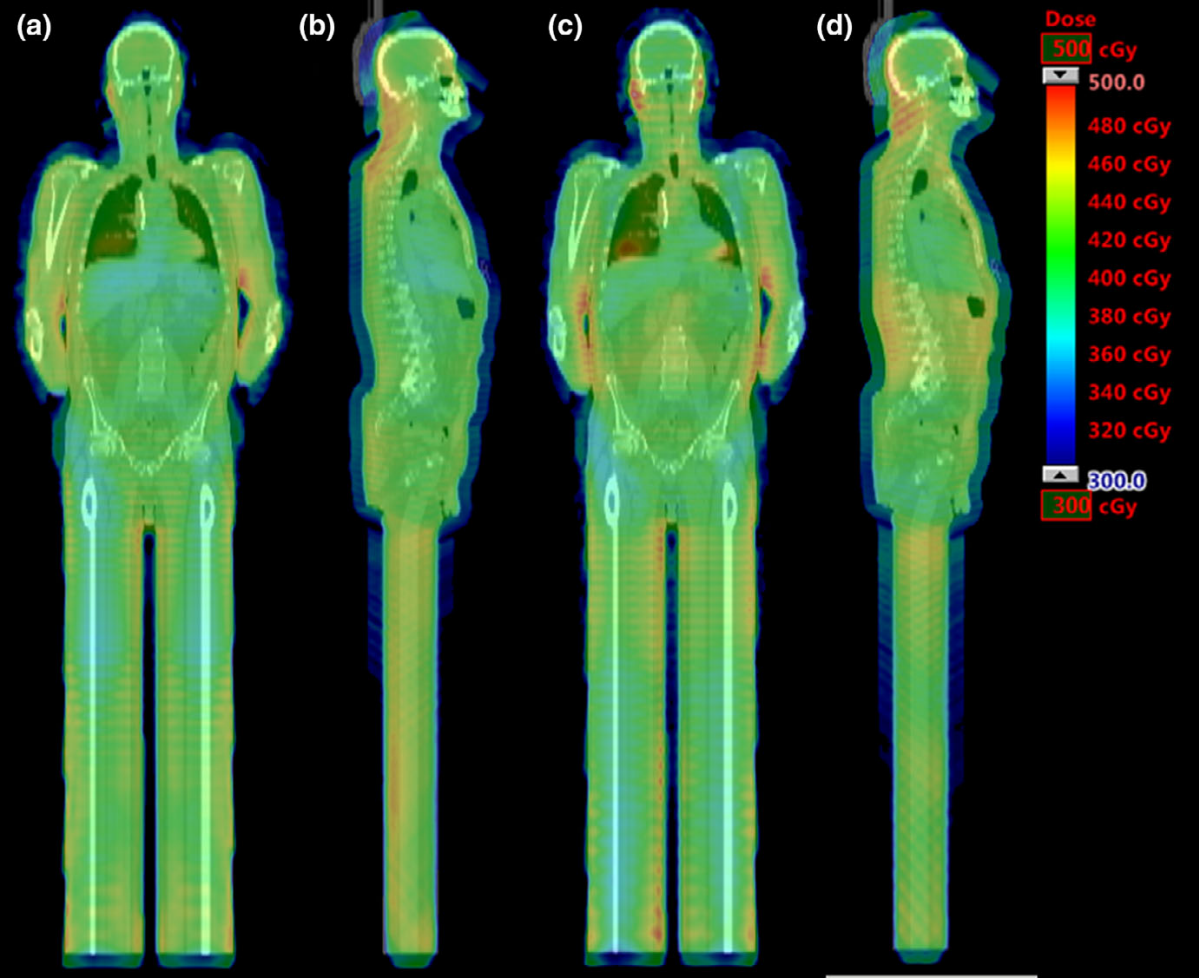
CT data of single coronal and sagittal slices from the same patient in the 21‐23 cm Tall categorization with dose color washes corresponding to each TBI plan. (a) and (b) show the clinical plan dose wash (clinicalTBI), and (c) and (d) the standard FFF plan dose wash (fffTBI).

**Figure 8:**
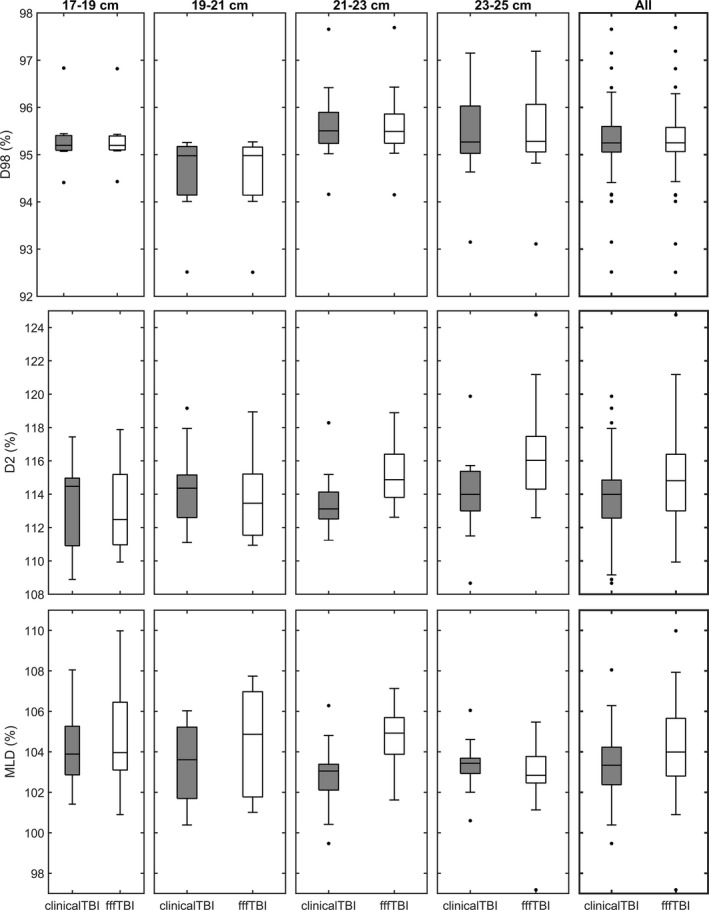
The dose‐volume histogram (DVH) parameter distributions of the clinical (clinicalTBI) and FFF (fffTBI) TBI plans for each patient categorization. The DVH parameters shown for all categories include the Body–5 mm D98% (top) and D2% (middle), and mean lung dose (MLD) (bottom). Each box moving left to right corresponds to the AP width categories in increasing order, ending with the data for all patients.

An example of CT data from one patient with the fffTBI and clinicalTBI in dose color wash in the coronal and sagittal planes is shown in Fig. 7. Qualitatively, the dose distributions vary between the two plans, particularly when examining hotspot location and lower dose regions. To quantitatively compare the plans with respect to dose homogeneity and lung dose, the D98% and D2% of the Body–5 mm structure and MLD were collected for both plans and all 47 patients. Uniformity is also reported via the homogeneity index. The resulting distributions are summarized in Fig. 8 and Table 3 for each separate patient category. Values are quoted as a percentage of the prescription dose where appropriate. All fffTBI and clinicalTBI were deemed clinically acceptable by the radiation oncologists. A majority of patients achieved our three ideal dosimetric criteria with fffTBI. Thirty‐eight patients met Body–5 mm D98% ≥95% with fffTBI, compared to 37 patients for clinicalTBI. For Body–5 mm D2% ≤115%, 26 patients met this with fffTBI, and 37 with clinicalTBI. Thirty patients achieved MLD ≤105% with fffTBI, and 40 with clinicalTBI. Patients with increased AP width did not meet the dose homogeneity goal as consistently, illustrated by the increased dissimilarity between the fffTBI and clinicalTBI Body–5 mm D2% distributions (Fig. 8). Of the 17 patients outside of MLD clinical tolerance with fffTBI, 11 were also outside of the Body–5 mm D2% tolerance. The only category‐specific significantly different fffTBI and clinicalTBI DVH distributions occurred in the 21‐23 cm Short category for the Body–5 mm D2% and MLD (Table 3). Across all of the categories, the differences between fffTBI and clinicalTBI medians for the Body–5 mm D2% and MLD were also significant (Table 3). In general, for all patient categories, the Body–5 mm D2% and MLD distributions for fffTBI were broader and shifted higher than those for clinicalTBI.

**Table 3:** Summary of dose‐volume histogram parameters for each patient categorization and both the clinical (clinicalTBI) and FFF (fffTBI) TBI plans.

Patient category	DVH parameter	clinicalTBI median (%)	fffTBI median (%)	clinicalTBI range (%)	fffTBI range (%)	*P*‐value
17‐19 cm	D98%	95.20	95.20	94.41‐96.83	94.43‐96.82	1.000
	D2%	114.5	112.5	108.9‐117.4	109.9‐117.9	0.721
	MLD	103.9	104.0	101.4‐108.0	100.9‐110.0	0.798
19‐21 cm	D98%	94.98	94.98	92.52‐95.26	92.51‐95.27	1.000
	D2%	114.4	113.5	111.1‐119.2	110.9‐118.9	0.734
	MLD	103.6	104.9	100.4‐106.0	101.0‐107.7	0.308
21‐23 cm Short	D98%	95.62	95.62	95.02‐97.66	95.03‐97.69	1.000
	D2%	112.6	115.0	111.3‐114.1	112.6‐117.6	**0.010**
	MLD	102.7	105.4	100.4‐103.4	103.9‐107.1	**0.0002**
21‐23 cm Tall	D98%	95.29	95.29	94.16‐96.27	94.15‐96.27	0.970
	D2%	113.8	114.9	111.3‐118.3	113.0‐118.9	0.121
	MLD	103.3	104.0	99.47‐106.3	101.6‐106.0	0.521
23‐25 cm Short	D98%	95.37	95.40	93.15‐97.15	93.11‐97.19	0.937
	D2%	113.8	115.2	108.7‐115.7	112.6‐124.8	0.310
	MLD	103.3	103.4	100.6‐103.6	102.6‐105.5	0.589
23‐25 cm Tall	D98%	95.17	95.16	94.63‐96.32	94.82‐96.29	1.000
	D2%	114.0	116.3	112.9‐119.9	114.0‐121.2	0.151
	MLD	103.7	102.4	102.0‐106.0	97.18‐105.1	0.222
All	D98%	95.2	95.3	92.52‐97.66	92.51‐97.69	1.000
	D2%	114.0	114.8	108.7‐119.9	109.9‐124.8	**0.041**
	MLD	103.3	104.0	99.47‐108.0	97.18‐110.0	**0.043**
	HI	1.20	1.20	1.14‐1.27	1.16‐1.28	0.088

Note

D98% and D2% both correspond to the Body–5 mm structure, and mean lung dose (MLD) corresponds to the mean dose to the lung structure. The homogeneity index (HI) is the ratio of the D2% and D98%. *P*‐values indicating significantly different medians (α = 0.05) are bolded.

### 3.2 Plan parameters

The number of MUs planned per arc for the supine and prone orientations were summed to give the total MUs per fraction for fffTBI and clinicalTBI. Beam‐on time was deduced based on the gantry speed at all control points over all arcs. The average dose rate at mid‐plane was the prescribed dose divided by the beam‐on time. The instantaneous dose rate at mid‐plane directly under isocenter is ~250‐300 and ~100‐130 cGy/min for fffTBI and clinicalTBI respectively, and is dependent on the patient AP width and MLC modulation. MU, beam‐on time, and average dose rate results from all 47 patients are listed in Table 4. These data are representative of that for each patient category examined separately. As the patient AP width increased, the MUs and beam‐on time increased, and average dose rate decreased at the same rate for both fffTBI and clinicalTBI. In comparing fffTBI to clinicalTBI, the MUs and average dose rate increased by 41.8% and 64.8% on average respectively, and the beam‐on time decreased by 39.2% on average.

**Table 4:** Various plan parameters including all patient categories for the clinical (clinicalTBI) and FFF (fffTBI) TBI plans.

Plan parameter	clinicalTBI median	fffTBI median	clinicalTBI range	fffTBI range
Average dose rate at mid‐plane (cGy/min)	11.07	18.07	9.85‐12.24	15.78‐20.66
Monitor units per fraction (MU)	10837	15496	9803‐12184	13552‐17744
Beam‐on time per fraction (min)	18.06	11.07	16.34‐20.31	9.68‐12.67

As alluded to in Section 2.B.2, the only difference in treatment planning time between the fffTBI and clinicalTBI is the time it takes to optimize the clinicalTBI. The amount of planning time saved by using standard plans was investigated by timing this step for ten patients, resulting in a median (range) time of 34.1 min (31.3‐41.4).

### 3.3 Plan performance with set‐up errors

Systematic set‐up errors were modeled in Eclipse by shifting arc isocenters of the fffTBI for 18/47 patients. Differences between the Body–5 mm D98% and D2%, and MLD for the shifted and original plans are summarized in Table 5 for all 18 patients. No trends across patient categories were seen. For shifts in the lateral and cranial‐caudal direction, the median dosimetric effect was less than or equal to 1.4%. Coverage tends to decrease and hotspots increase with both lateral shift directions as the MLC shielding is offset. The fffTBI are less sensitive to cranial‐caudal shifts with respect to D98% and D2%, but MLD changes are more pronounced with shielding being moved off of the lungs in the superior‐inferior direction. The maximum dosimetric discrepancies were seen with anteroposterior shifts that simulate an SSD mismatch. Changes of 2.9%‐5.0% in all DVH parameters are apparent in this case.

**Table 5:** Percent difference between the fffTBI DVH parameters and the systematically shifted DVH parameters.

Direction	DVH parameter	Shifted plan—original plan median (%)	Shifted plan—original plan minimum (%)	Shifted plan—original plan maximum (%)
Lateral patient right	D98%	−0.8	−3.0	0.6
	D2%	0.7	−0.5	2.3
	MLD	0.6	0.2	1.0
Lateral patient left	D98%	−1.2	−2.6	1.0
	D2%	1.4	−1.0	2.0
	MLD	0.1	−0.4	0.5
Anterior	D98%	−3.2	−3.6	−3.1
	D2%	−4.1	−4.6	−3.6
	MLD	−3.2	−3.4	−2.9
Posterior	D98%	3.4	3.1	3.7
	D2%	4.4	3.9	5.0
	MLD	3.4	3.1	3.6
Inferior	D98%	−0.1	−1.2	0.1
	D2%	0.1	−0.4	1.0
	MLD	−1.2	−1.9	−0.4
Superior	D98%	−0.1	−0.5	0.6
	D2%	0.3	−0.4	0.8
	MLD	1.4	0.2	2.1

Note:

D98% and D2% both correspond to the Body–5 mm structure, and mean lung dose (MLD) corresponds to the mean dose to the lung structure.

## 4 DISCUSSION

In this work, we have presented standardized TBI treatment plans developed from our clinical VMAT TBI technique that make use of FFF beams and their accompanying increased dose rate, this being the first attempt at using FFF beams in TBI treatment planning that we are aware of. The FFF VMAT TBI treatment plans (fffTBI) used a sweeping arc delivery that accounts for ISL effects and beam spoiler attenuation as per the clinical VMAT TBI technique. Lateral dose profiles in conjunction with RAOFs were used to calculate MLC leaf positions to yield a more homogeneous dose and shield the lungs. Six sets of supine and prone standardized MLC modulations were planned, and patients were assigned a plan based on AP width and height.

The change from using a standard linac beam with the flattening filter inserted to a FFF beam caused variations in the treatment plan dose distribution, as expected with a change in dose profile (Fig. 7). Even with the modified dose distribution, the same dosimetric criteria are generally met using either treatment plan. The Body–5 mm D98% had similar distributions for the fffTBI compared to the clinicalTBI because of plan normalization. Overall, the Body–5 mm D2% and MLD are significantly higher for fffTBI across the patient cohort, due to the use of a standardized plan instead of an optimized plan tailored to a specific patient. This is also evident in the increase in distribution range for fffTBI in all patient categories, as the modulation required for homogeneity in certain areas for one patient may not be as applicable to others in a given category, especially as the patient AP width increases. For those patients where the dosimetry is not ideal, it is possible to utilize Eclipse VMAT optimization as done for the clinical plan creation to achieve better plans.^8^ Using the last step of PRO for VMAT planning in Eclipse, where only the plan's MUs and MLC leaf positions can be modified, the fffTBI for the given patient AP width can be used as the optimizer's base dose plan to attain the additional MLC leaf motion required to meet uniformity and MLD goals. FFF optimization has been tested on six patients from the 23‐25 cm Short and Tall categories that did not meet dosimetric criteria, with all patients achieving the desired conditions post‐optimization. It has additionally been performed on patients with AP widths not considered for the current planning study up to the maximum AP width seen of 37.4 cm, where the standard 23‐25 cm plans were used to generate the base dose plans for optimization. These optimized FFF VMAT TBI plans met criteria or matched the corresponding clinical plan DVH. This illustrates that optimization using FFF beams could indeed be performed for patients as long as a standard FFF plan has been created, however it is not necessary in all cases and the reduced planning time would be lost. Optimizing the plans requires an additional 34 min that would not be needed when using the standard FFF plans alone.

The dose homogeneity decreased and MLD increased for the 21‐23 cm group when utilizing fffTBI. In addition, higher D2% values were seen for the 23‐25 cm group although they were not statistically significant. This trend over increasing patient AP width is likely related to patient separation, greater body contour variation across each category, and the beam energy being used. In this work, 6 MV both with and without a flattening filter was examined for ease of comparison as this is the energy used clinically, but the TrueBeam linacs (Varian Medical Systems, Palo Alto, CA) are capable of using 10 MV FFF for VMAT treatments. Using a higher energy for VMAT treatments results in additional patient neutron dose compared to 6 MV FFF VMAT plans, which is an area we wish to investigate in the future with respect to 10 MV FFF VMAT. Furthermore, the dose profile of 10 MV FFF is more peaked than 6 MV FFF, meaning that plans using 10 MV FFF would likely need even more MLC modulation to achieve the required dose homogeneity. Highly modulated plans are impacted more by set‐up errors, and would not take full advantage of the increased dose rate. It is therefore unlikely that moving to a higher energy would be advantageous for the patient cohort in this work. However, 10 MV FFF plans may provide an overall benefit for patients with AP widths greater than what was examined here.

It is expected that patient set‐up would be more critical when using FFF beams due to the accompanying increase in dose profile variation. In the case of TBI treatments, this is an especially important consideration as they often occur at an extended SSD without the use of image guidance. The dosimetric impact of having a large set‐up error of 2 cm was at most 3.0% for the lateral and cranial‐caudal directions. An SSD mismatch was more substantial, with changes between the shifted and original FFF plans being up to 5.0%. This indicates that care must be taken when moving the patient to the correct SSD. Similar trends were seen when examining the plan robustness to set‐up uncertainties for our current clinical technique.^8^ Overall, the fffTBI shows unanticipated robustness to set‐up errors, which is likely due to the MLCs decreasing the FFF dose profile peak and essentially flattening the dose profile.

The ultimate goal of using FFF beams in TBI VMAT treatment planning was to reduce treatment time to increase patient comfort. This objective would theoretically be achieved, with an average decrease in beam‐on time of 39.2% when moving to FFF treatment plans (Table 4). The time the patient spends in TBI treatment is not solely beam‐on time however; there are additional set‐up procedures to those often seen for conventional radiation therapy that require supplementary measurements that make up the majority of the scheduled time on the linac. At the TBCC, TBI appointments are one hour to account for set‐up and protracted beam‐on times, though a full hour is not necessarily required. Even though less than half of the booked time is beam‐on time (Table 4), the time savings seen in changing modality could result in appointment time reassessment and possibly free up resources to be used for other treatments. It is also important for patients as they would still be spending less time on the treatment bed. Increasing their comfort could result in reduced intra‐fraction motion as well, improving dose delivery accuracy. In addition, specifically moving to standardized treatment plans removes strain from planning resources since plan optimization is no longer required. Reported times for clinicalTBI treatment planning are 90‐120 min,^8^ so a time reduction of at least 26% is possible. Furthermore, standard plans require less repeated quality assurance (like portal dosimetry) to ensure that they are deliverable, thus freeing up even more time on treatment units. Though the work presented here strictly examined the feasibility of using FFF beams in TBI treatment planning and planning system reported beam‐on times, quantitatively investigating the associated changes in time efficiency at multiple stages of clinical TBI treatments is an area requiring further investigation.

Historically, TBI treatments have been performed with the lowest feasible dose rate as dose rate is thought to be related to potentially lethal complications that patients experience during HSCT conditioning and posttreatment. In particular, there has been work performed in the literature that investigates the relationship between dose rate and the incidence or lethality of pulmonary complications such as interstitial pneumonitis (IP) or radiation pneumonitis (RP). There has been prospective and retrospective evidence illustrating both an apparent dose rate effect,^10^, ^11^, ^12^, ^13^, ^14^ and no statistically significant dose rate effect.^15^, ^16^, ^17^, ^18^, ^19^ When considering the dataset as a whole, several problems are evident. First, a majority of the studies are retrospective, meaning that confounding variables and biases carry more weight. There was one prospective study completed by Ozsahin et al comparing different instantaneous dose rates for single‐dose and fractionated TBI schemes and their influence on a number of complications for 157 patients.^16^ For any combination of their TBI schemes including instantaneous dose rates between 3 and 15 cGy/min, no significant relationship between IP incidence and dose rate could be found, though the authors do note that their clinical experience suggested that there may still be an effect.^16^ Even though this work was prospective, it was constrained by a second, related problem that also plagues the literature: the number of confounding variables to account for in HSCT procedures. For HSCT regimens including TBI, factors like radiation prescription dose, fractionation, total lung dose, organ shielding, patient and beam orientation, beam energy, and dose rate must all be accounted for. In addition to these parameters, the patients' age, chemotherapy regimen, graft versus host disease (GVHD) prophylaxis, type of HSCT, disease, remission status, and performance status will all impact outcomes and complication incidences. Because the number of patients being treated per year at any particular institution is generally quite low,^3^, ^20^ accrual for both retrospective and prospective studies is often extended and thus the studies suffer from having patient cohorts with large variability in the parameters listed as techniques change over time. This variability is difficult to account for in statistical analysis. Determining the relationship between dose rate of TBI delivery and pulmonary complications is therefore a complex issue, and has not reached a consensus in the community. At the TBCC, we currently treat at much higher dose rates than those often used in published data (instantaneous dose rates of ~100 cGy/min versus ≤15 cGy/min respectively), and an internal retrospective analysis has found no instances of radiation pneumonitis. Clearly the pathogenesis of lung toxicity during HSCT is multifactorial and the role of radiation dose rate remains unknown.

A definitive relationship that has been acknowledged is that of pulmonary complications and total lung dose.^19^, ^21^ Lung shielding is often seen for both single‐dose and fractionated schemes to reduce lung dose and toxicity. Della Volpe et al have suggested MLD limits of approximately 9 Gy for a 10 Gy in three fractions and 5.5 cGy/min dose rate regimen to reduce the rate of lethal pulmonary complications to less than 5%,^22^ with this being the restriction many centers place on lung dose for a 10 Gy total body prescription currently. This is also in agreement with the lower incidences of lung toxicity for reduced‐intensity conditioning HSCT^23^ which generally uses radiation prescription doses that are less than those seen in conventional TBI, though the influence of reduced‐intensity chemotherapy on these toxicities must be recognized.

Though no patients have been treated with the fffTBI yet and there are no data on patient outcomes using this technique, we believe that high dose rate treatments for low‐dose TBI would be safe to perform due to the reasons outlined above. The TBCC's regimen results in lung doses that are far below those suggested to reduce radiation‐induced lung toxicity risk to an acceptable level, and modifying the treatment plans to FFF beams did not alter the MLD to a level of concern even though it was significantly higher for fffTBI (*P* = 0.043).

## 5 CONCLUSIONS

Presented in this work is an extended SSD VMAT TBI technique utilizing FFF beams, allowing a higher dose rate delivery. A simple methodology was developed to calculate MLC positions from RAOFs and lateral dose profiles for improved dose homogeneity. This led to the creation of standard VMAT TBI plans that are applied based on patient height and AP width at umbilicus that are able to achieve clinically acceptable dose homogeneity for most patients in the cohort examined. Standard plans reduced time spent in planning by 34.1 min per plan, which makes up approximately one third of our current planning time. In addition, the increased dose rate from the plans themselves decreased the beam‐on time by 39.2% compared to our current clinical technique. Overall, this new TBI FFF technique is theoretically feasible, robust to set‐up errors, and able to meet the same dose criteria as that achieved when using a flattening filter while improving time efficiency.

## CONFLICT OF INTEREST

The authors have no relevant conflict of interest to disclose.
